# Association of Self-Compassion With Suicidal Thoughts and Behaviors and Non-suicidal Self Injury: A Meta-Analysis

**DOI:** 10.3389/fpsyg.2021.633482

**Published:** 2021-05-28

**Authors:** Hanna Suh, Jisun Jeong

**Affiliations:** ^1^Psychology and Child & Human Development Academic Group, National Institute of Education, Nanyang Technological University, Singapore, Singapore; ^2^Graduate School of Education, Korea University, Seoul, South Korea

**Keywords:** self-compassion, protective factor, suicide, STBs, NSSI

## Abstract

**Objectives:** Self-compassion functions as a psychological buffer in the face of negative life experiences. Considering that suicidal thoughts and behaviors (STBs) and non-suicidal self-injury (NSSI) are often accompanied by intense negative feelings about the self (e.g., self-loathing, self-isolation), self-compassion may have the potential to alleviate these negative attitudes and feelings toward oneself. This meta-analysis investigated the associations of self-compassion with STBs and NSSI.

**Methods:** A literature search finalized in August 2020 identified 18 eligible studies (13 STB effect sizes and seven NSSI effect sizes), including 8,058 participants. Two studies were longitudinal studies, and the remainder were cross-sectional studies. A random-effects meta-analysis was conducted using CMA 3.0. Subgroup analyses, meta-regression, and publication bias analyses were conducted to probe potential sources of heterogeneity.

**Results:** With regard to STBs, a moderate effect size was found for self-compassion (*r* = −0.34, *k* = 13). Positively worded subscales exhibited statistically significant effect sizes: self-kindness (*r* = −0.21, *k* = 4), common humanity (*r* = −0.20, *k* = 4), and mindfulness (*r* = −0.15, *k* = 4). For NSSI, a small effect size was found for self-compassion (*r* = −0.29, *k* = 7). There was a large heterogeneity (*I*^2^ = 80.92% for STBs, *I*^2^ = 86.25% for NSSI), and publication bias was minimal. Subgroup analysis results showed that sample characteristic was a moderator, such that a larger effect size was witnessed in clinical patients than sexually/racially marginalized individuals, college students, and healthy-functioning community adolescents.

**Conclusions:** Self-compassion was negatively associated with STBs and NSSI, and the effect size of self-compassion was larger for STBs than NSSI. More evidence is necessary to gauge a clinically significant protective role that self-compassion may play by soliciting results from future longitudinal studies or intervention studies.

## Introduction

Suicide is one of the biggest public health concerns worldwide (Bachmann, 2018). Globally, 800,000 people die from suicide every year, and 1.4% of all premature deaths are suicides (World Health Organization, 2014). This trend has remained consistent over the past 50 years (Franklin et al., 2017), continuously calling upon researchers, practitioners, and policymakers to address preventing suicide and creatively intervening with those who are at risk for suicide. Suicidal thoughts and behaviors (STBs) produce wide and long-lasting effects, constantly increasing stress to mental health systems. The effects of STBs go beyond an individual, with negative psychological, social, and economic cascade effects propagating to surrounding social groups and larger communities and countries. Thus, significant prevention, intervention, and postvention efforts are being developed and tested constantly (e.g., World Health Organization, 2014).

There are many variations within suicidal thoughts and behaviors, including suicide-related thoughts, urges, preparatory acts, or attempts (Klonsky et al., 2016). O'Connor et al. (2013) outline differences within STBs. Suicide-related thoughts (ideation) include having either passive or active thoughts of ending life without preparatory behaviors. Suicide intent (urges) refers to the urge to want to die. Suicide-related thoughts and suicide intent may not always go hand in hand. For example, someone might experience thoughts of ending his/her life but may not want to die. Preparatory behaviors refer to activities involved in planning toward suicide. Last, a suicide attempt is a non-fatal self-directed injury with an intent to die, and suicide is a fatal self-directed injury with the intent to die (Crosby et al., 2011). Each of these STBs reflects different levels of severity (and correspondingly increasingly intensive intervention care) and depending on whether transient or chronic mental health conditions co-occur, the level of intervention also differs. However, a common basis of STBs is the desire to escape from psychological and physical pain.

A closely related, yet distinct, behavior from STBs is the non-suicidal self-injury (NSSI). The NSSI refers to engaging in self-destructive behaviors (e.g., cutting, burning, and punching) without the intention to die (Nock et al., 2006). Approximately 20% of young adults have a history of NSSIs (Swannell et al., 2014), and 70% of adolescents in clinical settings have reported both a history of NSSI and a suicide attempt (Nock et al., 2006). The reasons behind engaging in NSSI vary, but it is often when individuals desire to escape from negative emotional states (Bentley et al., 2014). Generally, no suicidal intent is present when engaging in NSSI, although research has shown that NSSI increases the possibility of suicidal behavior (Andover et al., 2012) such that future suicide attempts were associated with past NSSI (Bryan et al., 2015).

One mechanism by which repeated NSSI increases the risk for suicide attempts might be through a behavioral reinforcement process. That is, repeated NSSI can become a negative reinforcement for individuals by achieving the desired consequences. For example, individuals can avoid negative emotions by paying attention to their physical pain and, thus, use NSSI as a strategy to cope with negative emotions (Joiner, 2005; Hasking et al., 2017). Progressively repeated NSSI will habituate individuals to not fear physical pain, ultimately lowering psychological resistance to engage in lethal self-harm (Joiner, 2005), thereby increasing the likelihood of suicide attempts. Additionally, certain mental health conditions such as depression seem to function as a transdiagnostic risk and maintenance factor of both NSSI and suicide (Klonsky et al., 2013), corroborating that STBs and NSSI are closely related. Attending to these shared characteristics of STBs and NSSI, the Centers for Disease Control and Prevention (CDC) recently proposed that both can be classified as self-directed violence (Crosby et al., 2011). Thus, it is essential to explore whether any common underlying factors exist that are associated with both STBs and NSSI.

## Risk and Protective Factors of Suicidal Thoughts and Behaviors and Non-suicidal Self-Injury and Their Associations

Much prior research has focused on accurately identifying risk and protective factors associated with STBs and NSSI (see Franklin et al., 2017 for a review of STBs and Fox et al., 2015 for a review of NSSI). Internalizing psychopathology (e.g., anxiety, depression, hopelessness, and emotion dysregulation) and demographic variables (e.g., age, education, and employment) were two of the strongest risk factors for STBs, followed by externalizing psychopathology (e.g., aggressive behaviors and impulsivity), prior STBs, and social factors (e.g., abuse, family problems, isolation, peer problems, and stressful life events). Similarly, characteristics such as impulsivity, unpredictability, and hopelessness were strong risk factors for NSSI (Fox et al., 2015).

In conjunction, recent research on STBs and NSSI has focused on identifying protective factors. Beyond demographic variables, the most notable protective factors of STBs and NSSI include a sense of social belongingness (Marraccini and Brier, 2017), social support (Kleiman and Liu, 2013), feelings of hope (Jiang et al., 2020), and emotional intelligence (Cha and Nock, 2009) to name a few. In exploring these protective factors, it was highlighted that the presence of protective factors is not merely the absence of risk factors. For instance, not feeling hopeless does not automatically translate into feeling hopeful about self and future. In this vein, Franklin et al. (2017) emphasized that protective factors that are not a simple reversal of risk factors (e.g., no psychopathology or no alcohol use) should be set a priori to be explored. Identifying protective factors allows the intentional fostering of the cultivation of skills by interventions to increase these protective factors where applicable.

## Self-Compassion and Suicidal Thoughts and Behaviors and Non-Suicidal Self-Injury

A factor that may be negatively associated with both STBs and NSSI that is theoretically plausible for potential successful intervention is self-compassion. Self-compassion is operationalized as a healthy attitude toward oneself when experiencing life challenges and feelings of self-inadequacy (Neff, 2003, 2020). Researchers draw from philosophical and neuroscientific literature to conceptualize self-compassion. Neff (2003) referred to Buddhist teachings to draw connections between compassion for others and compassion for self, in which compassion refers to being touched by others' sufferings and pain and opening oneself to be connected to others' pain. Similarly, self-compassion was conceptualized as being connected with suffering and pain while sending warm acceptance to the experience of suffering of the self. Gilbert (2005) argued that being compassionate to others and self physiologically activates a soothing system (parasympathetic nervous system). In sum, self-compassion allows someone to be in touch with sufferings with kindness, providing a sense of soothing.

Self-compassion prepares a person to be less judgmental toward oneself, less isolated, and more balanced in perspectives so that he or she does not need be overwhelmed by negative emotions, mistakes, or failures (Neff et al., 2007). Because intense negative feelings and attitudes about the self (e.g., self-loathing or self-isolation) often accompany STBs and NSSI, self-compassion may counteract these negative emotional states. Indeed, self-compassion has been found to be negatively associated with STBs (Tielke, 2016; Rabon et al., 2018), and self-compassion has been found to moderate the effects of negative life events on STBs and NSSI (Jiang et al., 2016; Xavier et al., 2016; Chang et al., 2017; Hasking et al., 2019). With psychological and emotional well-being associated with STBs or NSSI, meta-analyses examining correlational studies have also found that self-compassion was inversely related to depression, anxiety, and stress (MacBeth and Gumley, 2012) and positively associated with psychological well-being (Zessin et al., 2015). Furthermore, considering that self-compassion could be cultivated through repeated practice, intervention studies have found that self-compassion-oriented interventions reduced psychological symptoms (e.g., anxiety, depression, eating behaviors, stress, rumination, and self-criticism) and increased life satisfaction and mindfulness (Ferrari et al., 2019; Wilson et al., 2019). In sum, empirical evidence points to exploring self-compassion as a viable factor that may be negatively associated with STBs and NSSI.

Two theoretical frameworks on STBs and NSSI, respectively, the interpersonal theory of suicide (IPTS; Joiner, 2005) and the cognitive–emotional Model of NSSI (CEM-NSSI; Hasking et al., 2017) can be extended to qualify the function of self-compassion. The IPTS argues that suicidal desires emerge when someone has a negative perception of oneself in relation to others (Joiner, 2005; Van Orden et al., 2010). Specifically, suicidal ideation is likely to emerge when an individual lacks a sense of belonging, accompanied by feelings of loneliness (thwarted belongingness) while perceiving him/herself to be a burden to others colored by self-hatred (perceived burdensomeness). Experiencing loneliness and feeling burdensome to others precisely reflects a lack of common humanity and being kind to oneself (Rabon et al., 2019). That is, self-compassion may reduce those self-defeating perceptions even in the face of mistakes and failures by understanding that feeling lonely is an inevitable aspect of human life, thereby allowing one to treat oneself with kindness.

The CEM-NSSI outlines that both emotion regulation and cognitive factors interactively influence NSSI, grounded in existing emotion regulation theories (Chapman et al., 2006) and social cognitive theory (Bandura, 1989, 1997). Specifically, emotional reactivity (e.g., temperament), representations of the self (e.g., “I am not worthy”), and NSSI-related cognitions (e.g., “cutting will provide immediate relief”) work synergistically, creating a vicious feedback loop that increases the risk for NSSI (Hasking et al., 2017). Holding a positive self-attitude (e.g., “I will be kind to myself”) can be introjected to weaken this negative feedback loop reducing self-judgment. Given the multiple developmental trajectories and risk factors that are associated with NSSI, Abdelraheem et al. (2019) recently emphasized the importance of finding modifiable factors that could introject or undermine the pathways and the effects of risk factors. After synthesizing 25 studies, they found that self-compassion, along with interpersonal difficulties, self-esteem, and impulsivity, can be a potentially modifiable factor. Modifiable factors can be targets for treatment, and much evidence suggests that self-compassion is a quality that can be cultivated through practice (Neff and Germer, 2013). In sum, self-compassion has the potential to counteract propositions of central aspects of both theories leading to STBs and NSSI.

To the best of the current knowledge, only one systematic review has explored the associative patterns of self-compassion with suicidal ideation and NSSI in 16 studies, finding that self-compassion was inversely associated with suicidal ideation or NSSI (Cleare et al., 2019). Although this review was informative, there were two limitations. First, Cleare et al. (2019) only focused on how self-compassion relates to suicide ideation and not any additional variations within STBs. Second, no meta-analysis was conducted, limiting an understanding of the relative importance of self-compassion against other factors.

This current study started with the goal to specifically address these two limitations. As such, first, we explored the relations between self-compassion and all STBs, expanding from a sole focus on suicide ideation in Cleare et al. (2019). Notwithstanding the qualitative differences in characteristics and the prevalence of each suicide-related variable, exploring all variants of suicide (ideation, thoughts, attempts, and plans) allows for the more comprehensive assessment of how self-compassion is related to STBs. Second, by conducting a meta-analysis, the current study derived effect sizes of correlations between self-compassion and STBs and NSSI. Quantifying effect sizes can be informative for future research when comparing the magnitude of their findings.

## Method

### Protocol and Search Strategy

**Figure 1:**
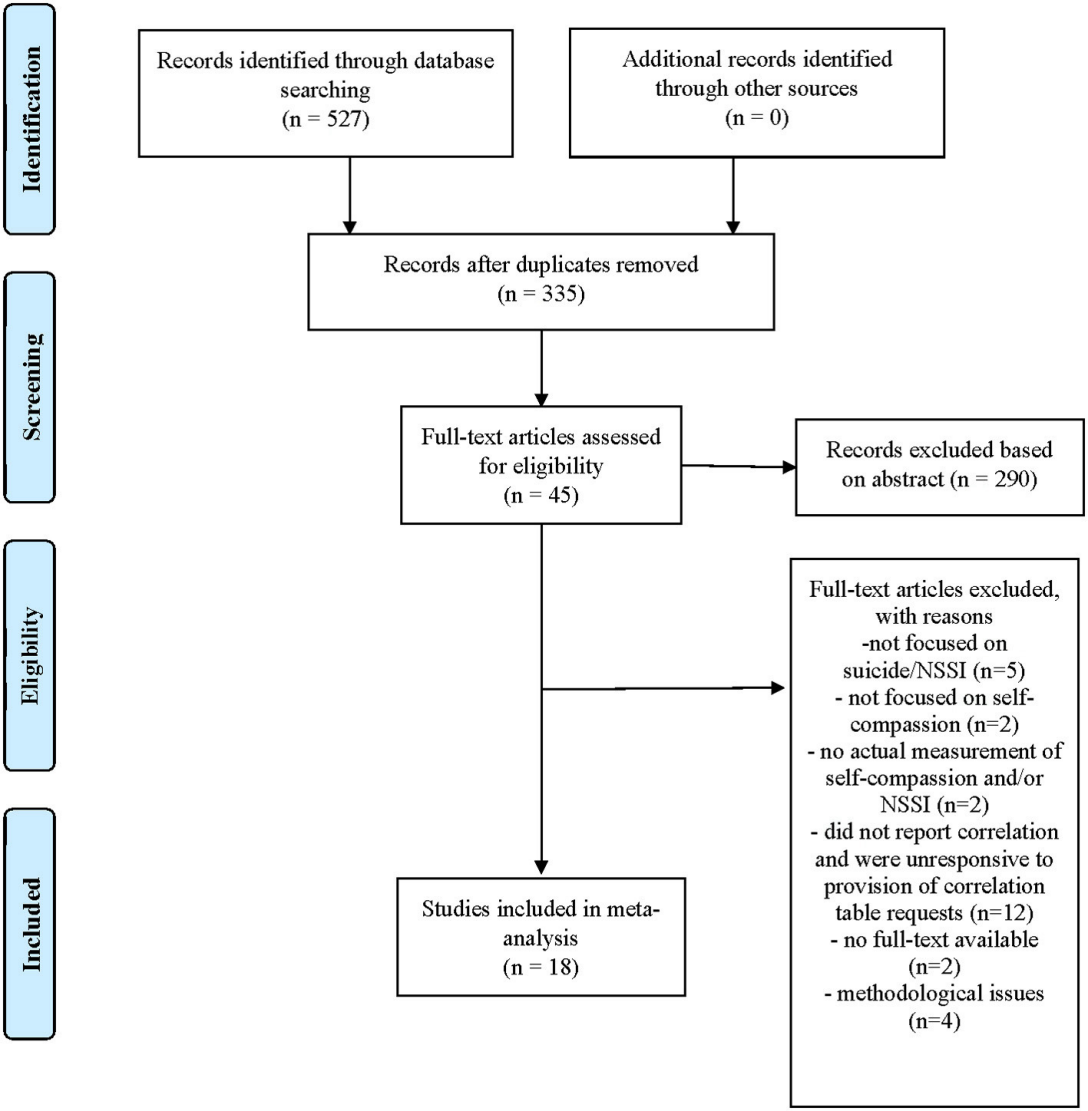
Flow diagram of the study selection.

This meta-analysis followed the Preferred Reporting Items for Systematic Reviews and Meta-Analyses (PRISMA) statement in conducting and reporting results (Figure 1). To begin, the first author conducted and finalized a systematic literature search on August 2, 2020, on five online databases (PsycARTICLES, ProQuest, PubMed, Web of Science, and PsycInfo) using search terms (mindful self-compassion or self-compassion) and (suic^*^ or self-harm or self-injury or self-mutilation or suicidal behavior or suicidal thoughts or suicidal ideation or cutting). Keyword searches were limited to titles and abstracts, and publications were from 2003 because the construct and measurement of self-compassion were introduced in 2003. Peer-reviewed articles, as well as theses and dissertations, were included in the initial search. Articles were limited to English articles. A total of 335 articles were initially identified after removing duplicate articles drawn from several databases, and the removal of duplicate articles was automatically done through the Mendeley reference management software.

### Inclusion and Exclusion Criteria

With 335 identified articles, the first and second authors separately screened the titles and abstracts for eligible studies. After this initial review, a total of 45 articles were identified for further full-text review to gauge whether they were eligible for final inclusion. Studies must have assessed self-compassion and at least one of STBs or NSSI to be eligible. The two authors independently excluded 290 articles. When there were discrepancies, they were cross-checked and confirmed until consensus was reached. Among 45 eligible studies, all studies measured self-compassion using the Self-Compassion Scale (SCS; Neff, 2003), which is the most frequently used measure of self-compassion. We looked for correlation reports, and corresponding authors were contacted for correlation results of the six subscales of the SCS, if not already provided in the manuscripts. Through email requests, seven provided the requested data (Xavier et al., 2016; LoParo et al., 2018; Hatchel et al., 2019; Sun et al., 2019; Wu et al., 2019; Zeifman et al., 2019; Vigna et al., 2020).

During the inclusion criteria review, 27 studies were excluded for the following reasons: studies did not focus on NSSI or suicide (*n* = 5), did not focus on self-compassion (*n* = 2), did not measure self-compassion and/or NSSI (*n* = 2), did not report correlation or were unresponsive to the provision of correlation table requests (*n* = 12), did not have available full-text (*n* = 2), or had methodological concerns (*n* = 4, detailed below). Published versions were used when articles were extensions of theses or dissertations (e.g., Vigna et al., 2020). There were no limits to sample type.

Regarding methodological concerns, one study (Tanaka et al., 2011) used dichotomous variable to assess suicide attempts (yes = attempted suicide, no = no history of suicide attempt). This methodology raised validity concerns in assessing STBs because it might inaccurately represent severity or frequency, as pointed out in their meta-analyses (Fox et al., 2015; Franklin et al., 2017). Hence, this study was excluded. Other studies used one or two items to assess STBs or NSSI, but these items were treated as ordinal variables with more than two response options (Jiang et al., 2016; Hasking et al., 2019; Hatchel et al., 2019; Wu et al., 2019; Sun et al., 2020; Vigna et al., 2020). For example, Hatchel et al. (2019) asked two questions to assess suicidal ideation and attempt, which were “During the past 30 days, have you seriously thought about killing yourself?” and “Have you attempted to kill yourself?” Such questions were treated as an ordinal variable with answer options of “No,” “Yes, but rarely,” “Yes, some of the time,” and “Yes, almost all of the time.” The current study used research that measured STBs or NSSI using one or two items when they had more than two response options because this allowed for frequency and/or severity differentiation. Additional subgroup analysis was conducted to compare the differences between questionnaire types (screening with one or two items vs. a validated whole measure). Notably, the current study included all suicide-related phenomena (suicidal attempt, ideation, plan, and behavior) to capture a comprehensive understanding of how self-compassion is related to STBs. Similarly, NSSI included all NSSI-related phenomena, including deliberate self-harm and frequency of NSSI.

Some studies were seemingly published using the same datasets: (a) Chang et al. (2017), Hirsch et al. (2019), and Kaniuka et al. (2019); (b) Jiang et al. (2016), Sun et al. (2020), and Wu et al. (2019); and (c) LoParo et al. (2018) and Sun et al. (2019). Specifically, Chang et al. (2017), Hirsch et al. (2019), and Kaniuka et al. (2019) seem to have used the same sample, as assessed by almost identical participant characteristics and correlation coefficients between STBs/NSSI with self-compassion. When the same dataset was used in multiple articles, the current study considered what correlates (STBs, NSSI) were measured and the details of the correlation coefficient report. First, for instance for (a) studies, Hirsch et al. (2019) and Kaniuka et al. (2019) were based on the same sample, but each study was on STBs and NSSI, respectively. Thus, both studies were included separately for STBs or NSSI. Furthermore, the current study included the correlation table from Hirsch et al. (2019) over Chang et al. (2017) because correlation results between self-compassion and STBs were more detailed (total score of the self-compassion scale was included). For (b) studies, Jiang et al. (2016), Sun et al. (2020), and Wu et al. (2019), the same sample seems to have been used as evidenced by the same research design (i.e., longitudinal study) and participant characteristics (e.g., Chinese adolescents). However, different dependent variables were reported: STBs (Sun et al., 2020) and NSSI (Jiang et al., 2016; Wu et al., 2019). Therefore, Sun et al. (2020) was included in the STB analysis. For NSSI, Wu et al. (2019) was included over Jiang et al. (2016) because the correlation table was more detailed. For (c) studies, LoParo et al. (2018) and Sun et al. (2019) appeared to have used the same sample, given the same intervention topic (i.e., cognitively based compassion training) and participant characteristics (e.g., African American suicide attempters). LoParo et al. (2018), who responded first to the correlation table request, was included.

### Data Extract

The second author coded studies on the following information: (a) mean age and standard deviation, (b) sample size, (c) sample characteristics, (d) type of self-compassion measure (short-form version vs. original long version), (e) type of STBs or NSSI measure, and (f) correlation coefficient. The first author and an independent reviewer (a master's level student) independently checked for data coding accuracy. Any inaccuracies were resolved before entered for analyses.

### Overall Data Analysis

Data were analyzed based on a random-effects model that assumed heterogeneity (Borenstein et al., 2009). Meta-analysis was performed using the Comprehensive Meta-Analysis (CMA) Version 3.0. In CMA, the *r* correlation coefficients were calculated into a pooled estimate by transforming to Fisher-Z and then converting back to *r*. According to Cohen (1988), effect sizes can be interpreted as *small* (*r* = 0.1), *medium* (*r* = 0.3), and *large* (*r* = 0.5).

When a study reported separate correlation coefficients for each subscale of the SCS instead of a composite score, a weighted average *r* (calculated by CMA) was utilized to generate an overall effect size of self-compassion. With longitudinal studies (Wu et al., 2019; Sun et al., 2020) that measured STBs or NSSI more than once, a weighted average *r* was entered. With studies (Hatchel et al., 2019; Sun et al., 2020) that reported separate results for suicide (e.g., suicide ideation and suicide attempt), a weighted average *r* was utilized to create one effect size between self-compassion and STBs. In cases where there were multiple correlates within a study, the effect size was separately analyzed per the correlate of either STBs or NSSI.

The Self-Compassion Scale (Neff, 2003) has three positively worded subscales and three negatively worded subscales (reverse code items). A high composite score of self-compassion indicates a high self-compassion tendency. To create a total score of SCS with six subscales following Neff (2003), the directionality of negatively worded scale coefficients (e.g., self-judgment, isolation, and overidentification) were reversed. For example, Kelley et al. (2019) reported a positive correlation between self-judgment and suicidality, *r* = 0.49. In this case, this was coded reversely as *r* = −0.49 when calculating a weighted *r* for the total SCS score.

### Quality Assessment

Quality assessment was conducted (Table 1). The National Institute of Health's 14-item quality assessment toolset for observational and cohort and cross-sectional studies was utilized. All 14 items were assessed, but eight criteria items that were relevant to the current meta-analysis were included: clarity on the research question and participation, missing data handling, inclusion and exclusion criteria, the rationale for sample size, valid and reliable independent and dependent measurement, and confound variable testing. The remaining six criteria items were excluded because these were only applicable to cohort studies. Each item was rated with three response options: Yes (0), No (1), or Other. The Other answer option could further be delineated into three reasons: cannot determine, not applicable, or not reported. Two independent raters (first and second author) conducted the quality rating of each study, and inter-rater reliability was derived. The interrater reliability for the quality rating of two raters was Kappa = 0.77 (*p* = 0.002), indicating substantial agreement between two raters (Landis and Koch, 1977). A “good” study indicated two or less weak components, and “fair” study indicated three weak components. The quality assessment results were used in meta-regression to explore sources of heterogeneity.

**Table 1:** Quality assessment results.

**Study**	**1**	**2**	**3**	**4**	**5**	**6**	**7**	**8**	**Mean**	**Overall rating**
**Suicidal behaviors**										
Collett et al. (2016)	0	0	NR	NR	0	0	0	1	1	Good
Hasking et al. (2019)^*^	0	0	0	CD	1	0.5	0.5	0	2	Good
Hatchel et al. (2019)	0	0	0	0	1	0	1	0.5	2.5	Fair
Hirsch et al. (2019)	0	0	0	0	1	0	0.5	0	1	Good
Kelley et al. (2019)	0	0	0	CD	1	0	0	0	1	Good
LoParo et al. (2018)	0	0	0.5	0	0	0	0	0	0.5	Good
Rabon et al. (2018)	0	0	0	CD	1	0	0	0	1	Good
Rabon et al. (2019)	0	0	NR	CD	1	0	0	0	1	Good
Sun et al. (2020)	0	0	0	0	1	0.5	0.5	0	2	Good
Umphrey et al. (2020)	0	0	NR	NR	1	0	0	1	2.5	Fair
Vigna et al. (2020)^*^	0	0	0	0	0	1	0	0	1	Good
Zeifman et al. (2019)	0	0	0	0	1	0	0	0	1	Good
Zhang et al. (2019)	0	0	NR	0	1	0	0	1	2	Good
**Non-suicidal self–injury**										
Forkus et al. (2019)	0	0	1	CD	1	0	0	1	3	Fair
Kaniuka et al. (2019)	0	0	0	CD	1	0	0	0	1.5	Good
Nagy (2017)	0	0	0	CD	0	0	0	0.5	0.5	Good
Wu et al. (2019)	0	0	0	CD	1	0	1	0	2.5	Fair
Xavier et al. (2016)	0	0	0	CD	1	0	0.5	0	2	Good

*Results show combined results of two assessors. (1). Was the research question or objective in this paper clearly stated? (2). Was the study population clearly specified and defined? (3). Was the participation rate of eligible persons at least 50%? (4). Were all the subjects selected or recruited from the same or similar populations? (5). Were inclusion and exclusion criteria for being in the study prespecified and applied uniformly to all participants? (6). Was a sample size justification, power description, or variance and effect estimates provided? (7). Were the exposure measures clearly defined, valid, reliable, and implemented consistently across all study participants? (8). Were the outcome measures clearly defined, valid, reliable, and implemented consistently across all study participants? (9). Were key potential confounding variables measured and adjusted statistically for their impact on the relationship between exposure(s) and outcome(s)?*

*Response options are 0 = Yes, 1 = No, or Other. Other could be delineated into CD, cannot determine; NR, not reported; NA, not applicable.*

* *Studies reported both suicidal thoughts and behaviors (STBs) and non-suicidal self-injury (NSSI)*.

## Results

### Study Characteristics

A total of 18 studies were included in the final analysis (see Table 2). Two studies were longitudinal studies (Wu et al., 2019; Sun et al., 2020), and others were cross-sectional. From 18 studies, 13 effect sizes were used for suicide, and seven effect sizes were used for NSSI. Because two studies (Hasking et al., 2019; Vigna et al., 2020) reported both STBs and NSSI, these studies were included for both STBs and NSSI analyses separately. For STB analysis (*k* = 13), a total of 5,989 participants were included, who were 56.23% women with a mean age of 29.96 (*SD* = 12.63). For NSSI analysis (*k* = 7), 4,124 participants were included, consisting of about half women (53.17%). The mean age was 20.94 years old (*SD* = 7.70).

**Table 2:** Study characteristics.

**Study**	**Sample *N***	**Self- compassion**	**Suicide or NSSI outcomes**	**Participants**	**Mean age (SD)**	**Female %**
**Suicidal behaviors**						
Collett et al. (2016)	42	SCS	BSS	Clinical participants with persecutory delusions, non-clinical participants	Clinical group 45.6 (12.1) Non-clinical group 41.9 (12.2)	52%
Hasking et al. (2019)	415	SCS-SF	Month ideation “In the last 12 months have you thought about ending your life?” Lifetime ideation “Have you ever thought about ending your life?”	College students	20.99 (5.33)	76%
Hatchel et al. (2019)	934	SCS-SF	Suicide ideation “During the past 30 days, have you seriously thought about killing yourself?” Suicide behavior “During the past 12 months, have you attempted to kill yourself?”	LGBTQ high school students	15.91 (1.18)	70%
Hirsch et al. (2019)	338	SCS	SBQ-R	College students	21.8 (5.3)	67%
Kelley et al. (2019)	189	SCS-SF	Subscale of IDAS	Veterans	43.14 (12.23)	5%
LoParo et al. (2018)	146	SCS	BSS	African American who had attempted suicide	42.4 (n/r)	53%
Rabon et al. (2018)	365	SCS-SF	SBQ-R	College students	21.44 (5.16)	66%
Rabon et al. (2019)	541	SCS-SF	SBQ-R	Veterans	49.9 (16.78)	31%
Sun et al. (2020)	520	SCS	Suicide ideation “Have you thought about suicide in the past 12 months?” Suicide Attempt “Have you attempted suicide in the past 12 months?”	Chinese adolescents	12.96 (2.33)	43%
Umphrey et al. (2020)	481	SCS-SF	CHRT	College students	29 (n/r)	71%
Vigna et al. (2020)	SI = 1,639 SA = 1,641	SCS-SF	Suicide ideation “Have you thought about suicide in the past 12 months?” Suicide Attempt “Have you attempted suicide in the past 12 months?”	Adolescents	n/r	58%
Zeifman et al. (2019)	130	SCS	SBQ-R	College students	21.04 (6.30)	83%
Zhang et al. (2019)	248	SCS	BSS	African American undergraduate students	37.26 (11.95)	56%
**Non-suicidal self-injury**						
Forkus et al. (2019)	203	SCS-SF	DSH	Veterans	35.08 (n/r)	23%
Hasking et al. (2019)	415	SCS-SF	ISAS	College students	20.99 (5.33)	76%
Kaniuka et al. (2019)	338	SCS	SHI	College students	21.81 (5.33)	67%
Nagy (2017)	72	SCS	ISAS	College students with a history of NSSI	19.37 (2.12)	n/r^*^
Vigna et al. (2020)	1,640	SCS-SF	“During the past 12 months, how many times did you do something to hurt yourself on purpose, without wanting to die, such as cutting or burning?”	Adolescents	n/r	58%
Wu et al. (2019)	813	SCS	Asking frequency of 12 NSSI behaviors “In the past 6 months, have you engaged in the following behaviors to deliberately harm yourself, but without suicidal intent?”	Chinese adolescents	13.15 (1.10)	43%
Xavier et al. (2016)	643	SCS	RTSHIA	Adolescents	15.24 (1.64)	52%

*BSS, Beck Scale for Suicide ideation (Beck and Steer, 1993); CHRT, Concise Health Risk Tracking Scale (Trivedi et al., 2011); DSH, Deliberate Self-Harm Inventory (Gratz, 2001); IDAS, Inventory of Depression and Anxiety Symptoms Suicide Scale (Watson et al., 2007); ISAS, Inventory of Statements About Self-Injury (Klonsky and Olino, 2008); RTSHIA, Risk-Taking and Self-Harm Inventory for Adolescents (Vrouva et al., 2010); SBQ-R, Suicidal Behaviors Questionnaire—Revised (Osman et al., 2001); SCS, Self-Compassion Scale (Neff, 2003); SCS-SF, Self-Compassion Scale—Short Form (Raes et al., 2011); SHI, Self-Harm Inventory (Sansone et al., 1998).*

* *No information on gender ratio for the participants with a history of NSSI (n = 72); n/r = not reported*.

### Self-Compassion and Suicidal Thoughts and Behaviors

**Figure 2:**
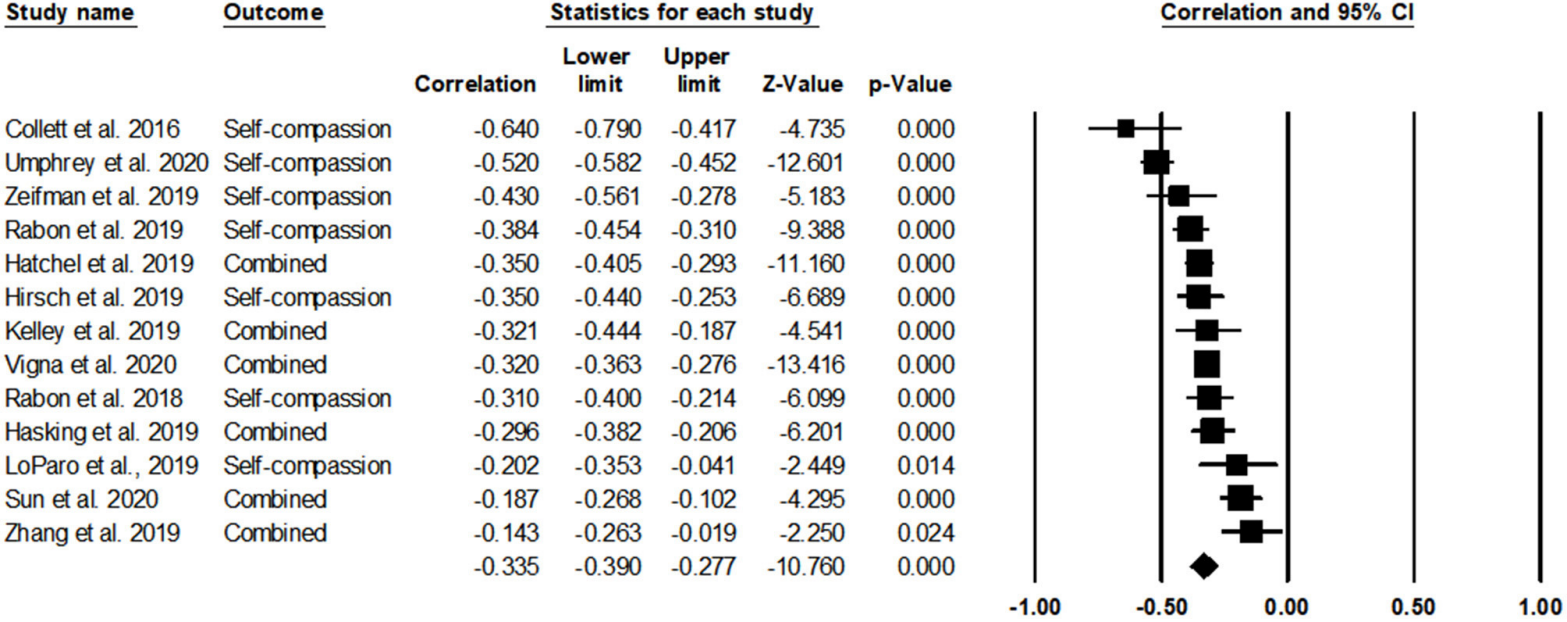
The association of self-compassion with suicidal thoughts and behaviors (STBs).

Table 3 and Figure 2 show the results of the meta-analysis. Note that studies labeled as “combined” indicate studies that reported individual subscale scores of self-compassion. The results indicated that self-compassion was significantly and negatively associated with STBs [*r* = −0.34, 95% CIs (−0.39, −0.28), *p* < 0.001]. In reviewing the forest plot for the contribution of each study on the calculation of overall effect size, three studies showed small effect sizes (*r*s = −0.20 to −0.14), eight studies showed moderate effect sizes (*r*s = −0.43 to −0.30), and two studies showed large effect sizes (*r*s = −0.64 to −0.52). Heterogeneity among studies was high, *Q*(12) = 62.89, *p* < 0.001, *I*^2^ = 80.92%.

**Table 3:** Effect sizes for composite and subscales of self-compassion.

**Correlate**	***k***	**Effect and 95% interval**			**Effect size classification**	**Heterogeneity**				**Test for overall effect**	
		**Effect**	**Lower**	**Upper**		***Q***	***df***	***p***	***I^**2**^***	***Z***	***p***
**Overall self-compassion**											
STBs	13	−0.34	−0.39	−0.28	Moderate	62.89	12	0.000	8.92	−1.76	0.000
NSSI	7	−0.29	−0.37	−0.20	Small	43.62	6	0.000	86.25	−6.39	0.000
**Self-compassion subscales**											
Self-kindness—STBs	4	−0.21	−0.37	−0.04	Small	2.07	4	0.000	8.07	−4.36	0.000
Self-judgment—STBs	4	0.11	−0.27	0.45	Small	91.27	3	0.000	96.71	0.56	0.579
Common humanity—STBs	4	−0.20	−0.27	−0.12	Small	3.98	3	0.264	24.52	−4.93	0.000
Isolation—STBs	4	0.13	−0.19	0.42	Small	64.06	3	0.000	95.32	0.80	0.423
Mindfulness—STBs	4	−0.15	−0.28	−0.01	Small	12.40	3	0.006	75.80	−2.09	0.037
Over-identification—STBs	4	0.07	−0.26	0.39	Small	7.28	3	0.000	95.73	0.42	0.676

The total score effect size results showed that self-compassion was negatively associated with STBs. To provide information on whether certain subscales of self-compassion showed stronger associations with STBs, each of the six subscales of the SCS was separately analyzed in relation to STBs. Of the 13 studies, six studies used the total score of the SCS. Four studies (LoParo et al., 2018; Hirsch et al., 2019; Zeifman et al., 2019; Zhang et al., 2019) reported or provided correlations of each subscale. Note that seven studies with the 12-item short-SCS were excluded for subscale analyses because the short-form recommends against using each subscale scores composed of only two items for each subscale (Raes et al., 2011). With four studies that reported subscale scores of the SCS, it was found that only positively worded subscales (self-kindness, common humanity, and mindfulness) exhibited statistically significant effect sizes: *r* = −0.21 for self-kindness, *r* = 0.20 for common humanity, and *r* = −0.15 for mindfulness. Negatively worded subscales did not show a statistically significant correlation with STBs.

### Self-Compassion and Non-suicidal Self-Injury

**Figure 3:**
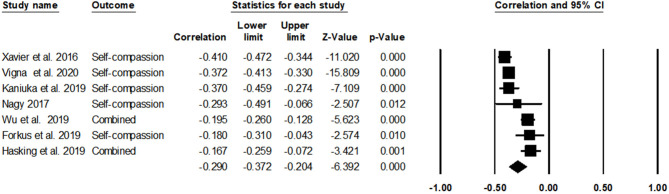
The association of self-compassion with non-suicidal self-injury (NSSI).

With seven studies, it was found that self-compassion was significantly negatively associated with NSSI [*r* = −0.29, 95% CIs (−0.37, −0.20), *p* < 0.001]. In reviewing the forest plot (Figure 3), four studies showed a small effect size (*r*s = −0.29 to −0.17), and three studies showed a medium effect size (*r*s = −0.41 to −0.37). Heterogeneity among studies was high, *Q*(6) = 43.62, *p* < 0.001, *I*^2^ = 86.25%. The associations between each subscale of SCS and NSSI could not be analyzed because there were only two studies that reported correlations with each of the six subscales.

### Possible Moderators

To probe potential sources of large heterogeneity, subgroup analyses were conducted to examine differences based on (a) sample type (marginalized identity sample, clinical patients, college students and healthy-functioning community adolescents, and past suicide attempters); (b) SCS questionnaire type (26-item SCS full version, 12-item SCS short version); and (c) STBs/NSSI questionnaire type (screening one or two items, a validated whole measure) (Table 4). None of the plausible subgroups had significant effects on STBs or NSSI except for the sample type with four categories on STBs. With STBs, effect size drawn from clinical patients [*k* = 1, *r* = −0.64, 95% CIs (−0.79, −0.42), *p* < 0.001] were stronger than sexually/racially marginalized sample [*k* = 3, *r* = −0.36, 95% CIs (−0.40, −0.32), *p* < 0.001] and college students and healthy-functioning community adolescents [*k* = 7, *r* = −0.35, 95% CIs (−0.43, −0.27), *p* < 0.001]. Participants with a history of a suicide attempt showed the smallest effect size among the four sample type categories [*k* = 2, *r* = −0.17, 95% CIs (−0.26, −0.07), *p* < 0.001].

**Table 4:** Moderation analysis results by population and measurement types.

	**Suicide behaviors**				**NSSI**			
**Correlate**	***k***	**Effect and 95% confidence**			***k***	**Effect and 95% confidence**		
		**interval**				**interval**		
		**Effect**	**Lower**	**Upper**		**Effect**	**Lower**	**Upper**
Population type	Between-group heterogeneity				Between-group heterogeneity			
	(*Q* = 2.63; *df* = 3, *p* = 0.000)				(*Q* = 2.39; *df* = 2, *p* = 0.303)			
Clinical patients	1	−0.64	−0.79	−0.42	0	-	-	-
Community sample	7	−0.35	−0.42	−0.27	5	−0.31	−0.40	−0.21
Past suicide attempters	2	−0.17	−0.26	−0.07	1	−0.29	−0.49	−0.07
Marginalized sample	3	−0.36	−0.40	−0.32	1	−0.18	−0.31	−0.04
SCS measurement	Between-group heterogeneity				Between-group heterogeneity			
	(*Q* = 0.80; *df* = 1, *p* = 0.372)				(*Q* = 0.50; *df* = 1, *p* = 0.479)			
SCS long version	6	−0.30	−0.42	−0.19	4	−0.32	−0.44	−0.19
SCS short version	7	−0.36	−0.42	−0.31	3	−0.25	−0.40	−0.08
Suicide/NSSI measurement	Between-group heterogeneity				Between-group heterogeneity			
	(*Q* = 1.35; *df* = 1, *p* = 0.245)				(*Q* = 0.00; *df* = 1, *p* = 0.980)			
Screening items	3	−0.28	−0.38	−0.18	2	−0.29	−0.45	−0.11
Validated measure	10	−0.36	−0.42	−0.28	5	−0.29	−0.40	−0.17

Furthermore, meta-regression analyses were conducted to address the high heterogeneity issue considering gender distribution (percent of female), sample size, and age (Table 5). The quality rating score was also entered to explore how much study quality affects effect sizes. None of the variables showed significant effects.

**Table 5:** Regression analysis results by age, gender ratio, sample size, and quality assessment rating.

	**Suicide**						**NSSI**					
**Correlate**	***k***	**Effect and 95% confidence interval**				***p***	***k***	**Effect and 95% confidence interval**				***p***
		**Effect**	***SE***	**Lower**	**Upper**			**Effect**	***SE***	**Lower**	**Upper**	
Age	12	0.00	0.00	−0.01	0.01	0.613	6	0.01	0.01	−0.01	0.02	0.551
Gender ratio (female %)	13	−0.14	0.17	−0.46	0.19	0.414	6	−0.15	0.32	−0.78	0.48	0.632
Sample size	13	0.00	0.00	0.00	0.00	0.867	7	0.00	0.00	0.00	0.00	0.425
Quality rating	13	−0.01	0.05	−0.11	0.09	0.871	7	0.09	0.06	−0.02	0.20	0.115

### Publication Bias

**Figure 4:**
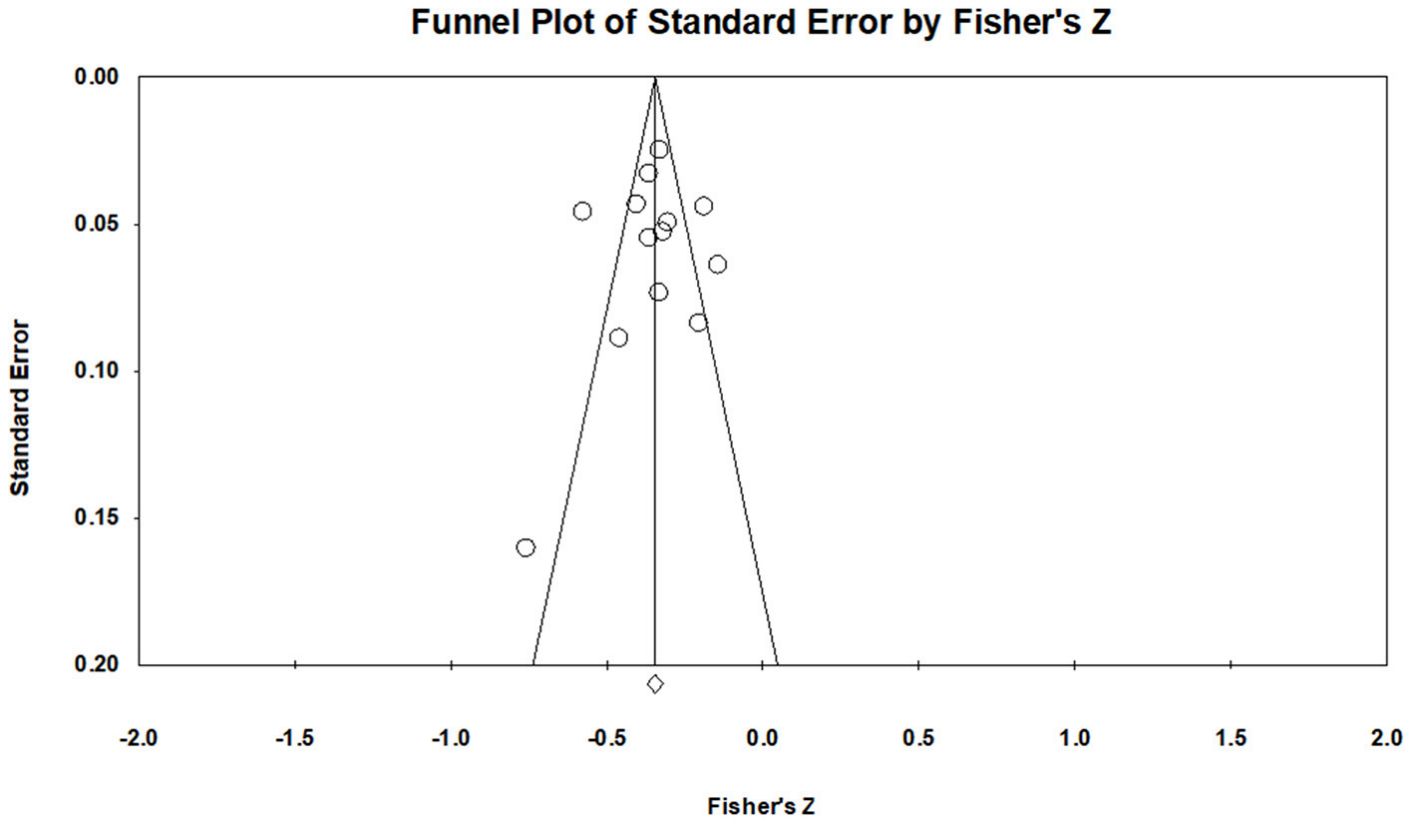
Funnel plots of STBs.

**Figure 5:**
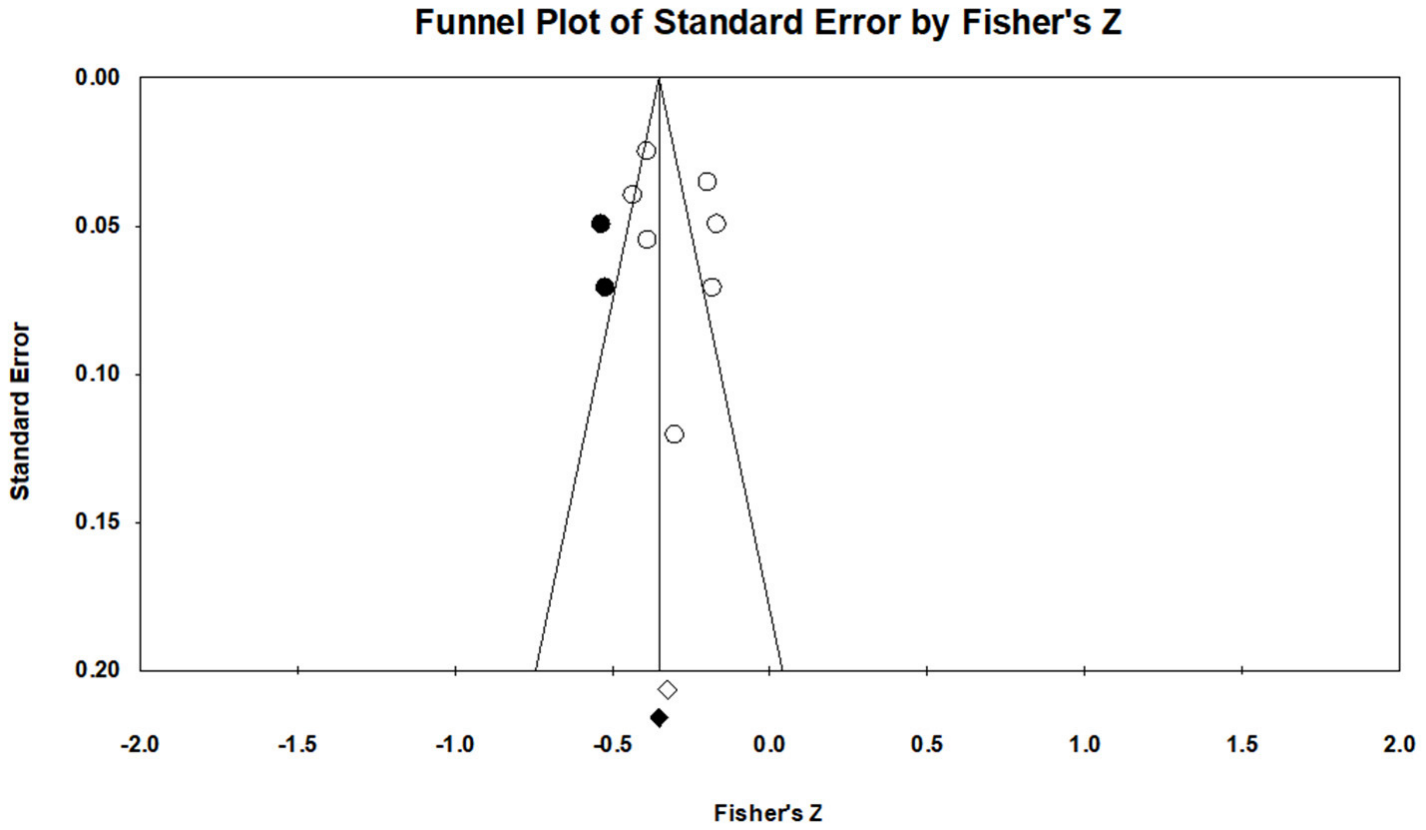
Funnel plots of NSSI.

To gauge publication bias, funnel plots were examined, and Egger's regression results were assessed. Publication bias refers to threats to the validity of the pooled effect size results from excluding unpublished studies (Rothstein et al., 2005). A funnel plot of STB studies suggested a slight trend of asymmetry (Figure 4). Therefore, additional quantitative analyses were conducted for the asymmetry of the funnel plot. The failsafe N for the STB variable was 2,069, indicating that at least 2,069 missing studies needed to refute the effect size's statistical significance. Egger's regression for the suicide variable was *p* = 0.772, indicating symmetry of a funnel plot. Thus, publication bias for STBs was deemed small. Visual investigation of publication bias for NSSI revealed a slight symmetry trend from the funnel plot (Figure 5). Additional quantitative analyses showed that the failsafe N for NSSI was 595, and Egger's regression was *p* = 0.439. In sum, results found that publication bias was minimal for NSSI.

## Discussion

This meta-analysis investigated whether and how self-compassion was associated with STBs and NSSI, examining studies published since 2003 when the construct of self-compassion was introduced and operationalized. A review of a total of 18 studies found that self-compassion was negatively associated with both STBs and NSSI, with moderate and small effect sizes, respectively. Furthermore, positively worded subscales of self-compassion were more strongly inversely associated with STBs, although a small sample size (*k* = 4) cautions against making definitive conclusions. These findings lend support to arguments positing that underactivation of a “positive pathway” (e.g., low self-compassion) should be considered in conjunction with the role of risk factors (e.g., high hopelessness) activating “negative pathway” toward suicide and NSSI (Chang et al., 2017). This dual-factor model of mental health (Wang et al., 2011) can account for the complex interactions of risk factors and (lack of) protective factors in understanding suicide and NSSI.

Self-compassion was negatively associated with STBs and NSSI. The medium and small effect sizes, respectively, are comparable to the effect sizes of other factors that are negatively associated with STBs and NSSI. For instance, school belongingness was negatively associated with suicidal thoughts and behaviors (OR = 0.54) (Marraccini and Brier, 2017), and engaging in physical activities was also negatively associated with suicidal ideation, such that physically active individuals were less likely to report suicidal ideation compared with those who were physically inactive (OR = 0.87) (Vancampfort et al., 2018). Furthermore, self-compassion was negatively associated with depression and shame (Johnson and O'Brien, 2013) and hopelessness (Zhou et al., 2013), all of which are risk factors for suicide (Davidson et al., 2011) and NSSI (Selby et al., 2012), with a large effect size of −0.54 reported in the association between self-compassion and psychopathology (MacBeth and Gumley, 2012). In sum, the role and effect of self-compassion appear comparable with other identified factors, and due to self-compassion's association with psychological distress that often co-occurs with STBs and NSSI, it is not surprising that self-compassion emerged as a significant factor negatively associated with STBs and NSSI.

While self-compassion is conceptualized as a trait with stable individual differences (rank-order consistency), the argument has been made that it is a skill that can be cultivated. Multiple randomized controlled trial results implementing self-compassion interventions across a wide array of individuals (individuals with depression, college students, mental health professionals, and adolescents) consistently found support for its effectiveness (Wilson et al., 2019; see for a review, Ferrari et al., 2019), with moderate effect sizes. For example, self-compassion-related therapies and interventions were effective in increasing self-compassion (*g* = 0.52; *g* = 0.75) and reducing anxiety (*g* = 0.46; *g* = 0.57) and depressive symptoms (*g* = 0.40; *g* = 0.66) (Ferrari et al., 2019; Wilson et al., 2019). Thus, implementing self-compassion interventions, which increase self-compassion, may be explored in the future as a potential factor to lower the likelihood of occurrence of and reduce the effects on STBs and NSSI.

Second, there were effect size differences between STBs and NSSI on the associations with self-compassion, such that the effect size of self-compassion was larger for STBs than NSSI. There may be several plausible explanations for this difference. One explanation might be that it is due to the different nature of STBs and NSSI. To elaborate, emotion dysregulation undergird the maintenance and exacerbation of both STBs and NSSI (Kranzler et al., 2016), but NSSI is additionally distinctively characterized by an uncontrollable urge for the action of self-injury. This characteristic makes NSSI essentially a behavioral problem (Nock et al., 2006), with emotional distress instigating this self-destructive behavior. Thus, with NSSI, attending to emotion dysregulation is important, and learning behavioral techniques to distract the action of NSSI is equally as important. Because self-compassion is associated with internal thoughts and emotions, as well as behaviors of NSSI, diffusion in the size of effect sizes might have led to an overall smaller effect size with NSSI compared with STBs. Indeed, other studies that report the association between self-compassion with behavioral indicators also show small effect sizes. For instance, in a meta-analysis that examined the association between self-compassion and health-promoting behaviors, a small effect size of *r* = 0.25 was shown (Sirois et al., 2015). On the contrary, studies that have examined the association between self-compassion with maladaptive thoughts and emotions showed large effect sizes (MacBeth and Gumley, 2012). In sum, NSSI having a behavioral component may be one plausible explanation for smaller effect size compared with STBs.

When comparing against each self-compassion subscale, positively worded (or “pure” self-compassion) subscales of self-compassion showed larger effect sizes than negatively worded subscales in the associations with STBs and NSSI. Multiple plausible explanations can exist. Our findings may reflect ongoing debates on the psychometric properties (and perhaps conceptual clarity) on what constitutes self-compassion and how it should be measured and calculated. If both factors equivalently reflected the general factor of self-compassion, it is unclear why such different effect sizes would emerge. Utilizing a large data of 11,685 participants, Neff (2020) recently found support for a single two-factor model (utilizing higher-order one factor of self-compassion) or a correlated six-scale model (utilizing all six subscale scores independently) to specify the SCS. The authors recommended against using correlated two factors of compassionate self-responding and non-compassionate self-responding, without a hierarchical one factor of self-compassion. However, our findings seem to lend support to distinguishing two higher-order factors with “non-compassionate self-responding” and “compassionate self-responding” with clearly distinctive associative patterns (direction of the association and the magnitude of association) between these two factors. As Neff (2020) mentioned, conceptualization and measurement of self-compassion ultimately is an empirical question that will become further clarified with accumulated evidence. Our findings lend support to distinguishing these two factors.

This differential associative pattern with suicide may be reflective of two different underlying systems that activate compassionate self-responding and non-compassionate self-responding (Muris and Otgaar, 2020). For instance, Gilbert and Kirby (2019) argued that (self) compassion physiologically activates the soothing system (parasympathetic nervous system), whereas non-compassionate responding activates the threat-defense system (sympathetic nervous system). Linking assessment of self-compassion to physiological measures, in addition to self-report assessment, to examine the activation and deactivation of the soothing system and the threat-defense system, could be one way to clarify the nature of self-compassion. At a minimum, three dimensions reflective of compassionate self-responding (mindfulness, common humanity, and self-kindness) appear to assess self-compassion reliably, and their associations with correlates such as STBs can also be reliably interpreted. This could be further supported through the comparable effect sizes of total SCS and positively worded self-compassion subscales with STBs.

There was high heterogeneity among studies on both STBs and NSSI. Exploration of plausible moderating variables (gender, types of SCS scales, original long vs. short version, types of suicide/NSSI scales, and study quality) did not significantly explain the heterogeneity. However, subgroup analysis results found that sample type was a significant moderator explaining heterogeneity in effect sizes. This is consistent with previous studies that show different severity levels of STBs and NSSI per sample characteristics. Those who were diagnosed with a psychiatric disorder and with past suicide attempts were more likely to report higher levels of STBs (Franklin et al., 2017). Those with psychiatric disorders were more likely to report NSSI (Fox et al., 2015). The STBs and NSSI are not prototypically expected responses to stressful experiences, and how individuals respond to stress depends on their current level of overall functioning. This explains why there was a moderating effect of sample type.

Regarding other tested moderating variables, the results on gender were surprising, especially given the rich literature on gender differences on STBs and NSSI. For STBs, previous studies show mixed results in the association between gender and suicide, such that men are at higher risk of suicide (Hedegaard et al., 2018), while other studies have found comparable risks between genders (Kaplan et al., 2012). Similarly, for the gender difference on NSSI, some studies have found that women are more likely to engage in NSSI (see for a review, Bresin and Schoenleber, 2015), while other researchers have found no differences in engagement between genders (e.g., Garisch and Wilson, 2015). With self-compassion alone, on the other hand, Yarnell et al. (2015) found that men showed higher self-compassion scores than women (*d* = 0.18) after a meta-analysis of 71 studies. Thus, we speculate that the effect of gender was offset due to mixed directionality on self-compassion, STBs, and NSSI by gender. For SCS scale type, when used in total score, the full SCS and short-SCS seem to yield comparable results, thereby showing a non-significant moderating effect. Except for a few studies, most studies used a validated measure of STBs (*k* = 10) and NSSI (*k* = 5), and most studies (14 of 18 studies) were rated as good quality, which may explain the nonsignificant moderating effect.

Multiple factors may explain the heterogeneity, although they were not testable within the current meta-analysis. One plausible explanation is that there was significant variation in social environments and conditions of survey administration (Imrey, 2020). For instance, there were significant geographical regional differences (e.g., different states within the United States, multiple countries worldwide, such as the United States, China, and Portugal). However, no theoretical and empirical support exists to hypothesize and test whether a certain regional and social condition would be predictive of a stronger association with STBs or NSSI than others. Furthermore, categorizing regions also seemed rather arbitrary, with difficulties determining whether state-level categorization (given that most studies were from the United States) or country-level categorization is appropriate. Another reason for high heterogeneity might be due to the nature of the correlate. High heterogeneity is common in meta-analyses that test the incidence or prevalence of a phenomenon (Imrey, 2020). Other meta-analyses on suicide and NSSI also reported high heterogeneity (Huang et al., 2017).

## Limitations and Future Directions

This meta-analysis has several limitations that may inform future studies on this topic. First, the sample size of this meta-analysis was small. Although the small sample size reflects the maturity of the self-compassion and STBs and NSSI literature, the results of this study suggest that more pointed future examinations could be conducted. For instance, examining the role of self-compassion in samples that are at high risk for STBs/NSSI (e.g., trauma clients and individuals with relapse of major depressive disorder) could be fruitful, given that self-compassion may be an especially difficult quality to embody among those who are psychologically persistently unhealthy. Relatedly, interpretations about the SCS subscale relations to STBs should be interpreted with caution because the results were based on four studies. Given that the psychometric properties of the SCS with six subscales or three positively worded subscales are an ongoing debate (Neff et al., 2019; Muris and Otgaar, 2020), future studies should specifically focus on exploring the nature of relations between SCS subscales and STBs/NSSI. Similarly, the moderating effect of sample type was significant, but because each level (four types) contained a small number of studies (e.g., clinical patients, *k* = 1), conclusions should be cautiously made. At a minimum, consistent with previous studies (Franklin et al., 2017), this study found that the psychological functioning of individuals is important to consider.

Second, all but two studies included in this meta-analysis were cross-sectional studies, limiting conclusions on whether self-compassion is a protective precursor to reduced STBs and NSSI. Conceptually, it is plausible that self-compassion is a causal protective predictor, but future studies should infuse longitudinal design and elucidate the nature of associations more in detail. Third, the delineation of different subtypes of suicide was unavailable in this meta-analysis because there were only two or three studies for each of the subtypes of STBs (e.g., suicide ideation, suicide thoughts, and suicide attempt). It was deemed inappropriate to summarize results from such few studies. Further accumulation of empirical studies on this topic would be necessary to draw generalizable claims. Fourth, all eligible studies could not be analyzed as the final sample because correlations were not reported and available in some studies. Contacting the authors for correlation tables was unsuccessful with a few studies, which prevents a more comprehensive convergence of scholarly evidence. Future studies could ensure the inclusion of basic descriptive statistics such as means, standard deviations, and correlations to advance the literature on self-compassion and STBs/NSSI at large.

Last, examining other variables in the association between self-compassion and STBs/NSSI was not conducted in this study, and it focused on establishing self-compassion as an important factor in STBs/NSSI. Future studies could examine mechanisms through which self-compassion affects STBs and NSSI. This would be an especially welcomed addition to the literature that potentially bolsters whether underactivation of “positive pathway” according to the dual-factor model is relevant to STBs and NSSI. An examination of other protective factors (e.g., sense of belongingness, hope, and optimism) in conjunction with self-compassion and comparing their relative strength of association with STBs and NSSI would be a fruitful area of future research.

Despite these limitations, this meta-analysis provides a useful starting point for future research to continue to explicate the nuanced associations between self-compassion and STBs and NSSI. Clinically, this study points to the possibility of infusing self-compassion interventions to increase self-compassion so that a protective pathway for STBs and NSSI could be considered, although more evidence should accumulate through longitudinal and interventions studies. Several group programs, such as the Mindful Self-Compassion (MSC) program (Neff and Germer, 2013) and C ompassion-F ocused T herapy (CFT; Gilbert, 2014), are carried out in clinical settings targeted to increase self-compassion and compassion. However, the degree of change per person has not been identified, nor whether these programs are similarly effective across varying degrees of psychopathology. Thus, more converging evidence of rigorous randomized controlled trials with at-risk populations for STBs and NSSI should be accumulated to recommend with confidence before self-compassion interventions can be more widely applied across different settings.

## Data Availability Statement

The original contributions presented in the study are included in the article/supplementary material, further inquiries can be directed to the corresponding author/s.

## Author Contributions

JJ designed and executed the review, performed data analyses, and wrote the paper. HS collaborated with analyzing data, wrote the paper, and edited the final manuscript. All authors contributed to the study.

## Conflict of Interest

The authors declare that the research was conducted in the absence of any commercial or financial relationships that could be construed as a potential conflict of interest.
